# 2025 Acknowledgment of *mSystems Ad Hoc* Reviewers

**DOI:** 10.1128/msystems.01706-25

**Published:** 2026-01-20

**Authors:** Ashley Shade

**Affiliations:** 1 Universite Claude Bernard Lyon 1, Laboratoire d'Ecologie Microbienne (CNRS UMR 5557), Villeurbanne, France

## EDITORIAL

For many scientists, it has been a year of rapid change and uncertainty in research. Despite these challenges, the expert reviewers of *mSystems* research articles have continued to show up and provide their critical service. Because these reviewers value the peer review system and its essential role in ensuring high-quality research is published, they devote their limited time and share their deep expertise in service of *mSystems* and the American Society for Microbiology. For this, we are incredibly grateful.

It is your dedication, high integrity, professionalism, and willingness to provide thoughtful, critical feedback to improve research that help keep *mSystems* at the forefront of systems microbiology research. This is an era in science defined by heightened cynicism about the academic publication ecosystem, with conversations dominated by ample stories of insubstantial or erroneous artificial intelligence-generated reviews and manuscripts, paper mills, monetized authorships, and predatory journals. And yet, research advances despite these hurdles, mainly due to the selfless motivation of researcher-experts who do their part to maintain the integrity of peer review.

Science relies on reviewers to be careful by scrutinizing details and asking direct questions that uphold scientific standards while exemplifying our values of fairness, measuredness, and (yes!) kindness. Early-career researchers learn from the examples that reviewers set. Established researchers gain key insights from reviewers’ expert perspectives. In the best cases, manuscripts are strengthened and new research directions are inspired.

Peer review remains an essential cornerstone for research. And as long as peer reviewers continue to serve with conviction, rigorous science will advance and offer value to society.

On behalf of *mSystems*, I sincerely thank the following individuals, who served as reviewers for the journal from December 2024 through November 2025.

Ruth Chingcuangco Abanador

Ghazanfar Abbas

Ghulam Abbas

Rehab Z. Abdallah

Mohamed Abdelmonem

Shairah Abdul Razak

Masahiro Abe

Hussein Adil Abid

Florence Abravanel

Monica Abrudan

Zaky Adam

Philip P. Adams

Kamoru A. Adedokun

Innocent Afeke

Surya D. Aggarwal

Somya Aggarwal

Rosa Aghdam

Augustine Kwaku Agyekum

Sarah Ahannach

Zakiah Anis Ahmad Nawawi

Tae-Hyuk Ahn

Christian H. Ahrens

Chunxai Ai

Ilya R. Akberdin

Yvonne Marvellous Akpudo

Alexander A. Aksenov

Mohammad T. Albataineh

Alexandra Alexiev

Qasim Ali Ali

Matthew Aliota

Celeste Allaband

Mohammadreza Allahyartorkaman

Alexander B. Alleman

Kylie Allen

Emma Allen-Vercoe

Fausto Almeida

Tania Alonso-Vásquez

Francis Alonzo

Ellinor Alseth

Tobias B. Alter

Ianina Altshuler

Sudheer Kumar Aluru

Samuel Alvarez-Arguedas

Andrew Alverson

Samat Amat

Ricardo Amils

Amnon Amir

Nana Ama Amissah

Alongkorn Amnuaykanjanasin

Mia Beatriz Cantera Amoranto

Kirk E. Anderson

Matthew Zack Anderson

Vijay C. Antharam

Josefa Antón

Masataka Aoki

Senu Apewokin

Hiroshi Arai

Michelle Aries Marchington

April Armes

Aman Ashar

Sarit Avrani

Taj Azarian

Andrea Azcarate-Peril

Sheyda Azimi

Taeok Bae

Rani Baes

Zeynep Baharoglu

Ren Bai

Tao Bai

Marcella Baiz

Frederik Bak

Craig Baker-Austin

Sophia Bałdysz

Yuanyuan Bao

Fernando Baquero

Bidisha Barat

John Barlow

Samuel Barnett

Blanca Barquera

Rodolphe Barrangou

Matthieu Barret

Jonelle T. R. Basso

Daniel A. Bastías

Joyoti Basu

Sulagna Basu

Tiffany Batarseh

Leo Baumgart

Denis Baurain

Abdulahad Bayraktar

Clifford J. Beall

Kimberly Elizabeth Beatty

Ashley E. Beck

Samantha L. Bell

Mia Bengtsson

Sarah Beno

Mikkel Bentzon-Tilia

Ivan Berg

Hanna Leah Berman

Marta Bermejo-Jambrina

Louis Berrios

Valérie Berthelot

Yogendra Bhaskar

Tamanash Bhattacharya

Jennifer F. Biddle

Eva Biggs

Jordan E. Bisanz

Stephanie L. Bishop

Alfredo Blakeley-Ruiz

Robert E. Blankenship

Alissa C. Bleem

Callan Bleick

Jon S. Blevins

Louis-Marie Bobay

Kasun H. Bodawatta

Robert A. Bonomo

Alessandra Borgognone

George Bouras

Patric Bourceau

Andrew Bridges

Vanessa Brisson

Robert A. Britton

Julie A. Brothwell

Bryan Paul Brown

Chris Brown

Heidi Brown

Titus Brown

Jeremy C. Brownlie

Holger Brüggemann

Pauline Bruyant

Jessica A. Bryant

Kate Buckeridge

Daniel H. Buckley

Nam Bui

Vinicius Buiatte

Michal Bukowski

Simon R. Bulman

Liana T. Burghardt

Alita R. Burmeister

James Butcher

Zibusiso Mayibongwe Buthelezi

Nina Bziuk

Sinem Çağlayan

Jingyi Cai

Lanlan Cai

Miaomiao Cai

Zhendong Cai

Antonio Camargo

Andrew D. S. Cameron

Ashley N. Campbell

Danielle Elizabeth Campbell

Shulin Cao

Gregory Caporaso

Rey Carabeo

Miguel Carda Diéguez

Allison F. Carey

Ross P. Carlson

Lisa A. Carmody

Michele Castelli

Santiago Castillo-Ramírez

Javier A. Ceja-Navarro

Jorge Cervantes

Kaan Çeylan

Ali Chaari

Jianmin Chai

Dipanjan Chakraborty

Patricia P. Chan

Josephine R. Chandler

Sriram Chandrasekaran

Rachael B. Chanin

Guillaume M. Charriere

John M. Chaston

Tiyakhon Chatnaparat

Harshali V. Chaudhari

Koel Chaudhury

Rongxiao Che

Casey Chen

Ce Chen

Chang Chen

Daijie Chen

Feifei Chen

Fusheng Chen

Guo-Qiang Chen

Haoyan Chen

Juanjuan Chen

Jun Chen

Lianmin Chen

Limin Chen

Litong Chen

Mingfei Chen

Nansheng Chen

Peigen Chen

Qinglin Chen

Qingxue Chen

Qiyi Chen

Qu Chen

Tingtao Chen

Tsute Chen

Wei-Hua Chen

Yan Chen

Yanfei Chen

Bisheng Cheng

Jiujun Cheng

Lei Cheng

Qiwen Cheng

Yuan-Bin Cheng

Zhongyi Cheng

Loo Wee Chia

Cynthia Maria Chibani

Katherine Chou

Ming-Yi Chou

Thomas Brian Clarke

Chaevien Clendinen

Tyler Coale

José Francisco Cobo Díaz

Emily Coffey

Arzu Çolak

Maureen L. Coleman

James Collins

Charles Coluzzi

Andre M. Comeau

Ciaran Condon

Chris Connor

Ryan Cook

Doug Cossar

Simone Raposo Cotta

Pauline M. L. Coulon

David Couvin

Don A. Cowan

Alexander Crits-Christoph

John Cronan, Jr.

John Cryan

Guzhen Cui

Jie Cui

Guzhen Cuiguzhen

Ashley L. Cunningham

Daniele Daffonchio

Paul M. D’Agostino

Ram Hari Dahal

Doralyn S. Dalisay

Robert E. Danczak

Thomas Dandekar

Maude David

Michael Z. David

Irene Davidova

Daniel Davila

Justine W. Debelius

Svetlana N. Dedysh

George S. Deepe

Kilian Dekoninck

Felipe Del Canto

Gerardo Della Sala

Álvaro Rodríguez Del Río

Daniel P. Depledge

Charlotte De Rudder

Matsapume Detcharoen

Hugo Devilliers

George C. Dicenzo

Katja Dierking

Natalie Diether

Lucia Diez-Gutierrez

Paul Dijkstra

Joseph P. Dillard

Natasha Dimova

Yousong Ding

Yuanzhao Ding

Choaro D. Dithugoe

Steven Philip Djordjevic

Stephen K. Dolan

Sara Domingues

Ning Dong

Anna Dongari-Bagtzoglou

Margaret Doolin

Sarah M. Doore

Blake Downing

Rebecca T. Doyle

Kevin Drace

Glen D’Souza

Hong Du

Benjamin Dubois

Quinten R. Ducarmon

Seána Duggan

Ilija Dukovski

Marc G. Dumont

Colum P. Dunne

Olivier Duron

Jonathan Dworkin

Michelle Dziejman

Marina Dziuba

Vonetta L. Edwards

Patrick Eichenberger

John Eid

Sofiane El-Kirat-Chatel

Mostafa S. Elshahed

Laramy Enders

Tobias Engl

Slava Epstein

Ana V. Espinel-Ingroff

Taryn Ashley Eubank

Marten Exterkate

Andrew James Fabich

Gloria Facklemann

Megan L. Falsetta

Yupeng Fan

Zhenxin Fan

Na Fei

Chenchen Feng

Hongjie Feng

Huili Feng

Limin Feng

Xiaowen Feng

Yingang Feng

Christopher Fenno

Isabel Fernández Escapa

Pamela Ferretti

Anna Fijarczyk

Alain Filloux

Marie Filteau

Monika S. Fischer

Theodore M. Flynn

Viacheslav Fofanov

Leandro Fonseca De Souza

Evan Forsythe

Jeffrey T. Foster

Michael J. Franklin

Claudia Frey

Florian Fricke

Elliot S. Friedman

Adrian Fritz

Ting Fu

Yingyi Fu

Yu Fukasawa

Takema Fukatsu

Julia Fukuyama

Hiromasa Funato

Kathleen Furtado

Uri Gabbay

Aida G. Gabdoulkhakova

Joao Gabriel Silva Souza

Yunn-Hwen Gan

Bing Gao

Guangliang Gao

Nan Gao

Rongsui Gao

Paul Gao

Shan Gao

Xiang Gao

Xuefeng Gao

Arkadiy I. Garber

Erin C. Garcia

Marc Garcia-Garcera

Inmaculada García-Romero

Daniel Rios Garza

Shaohua Ge

Zhongming Ge

Barney A. Geddes

Rebecca Gellman

Milan Gerovac

Rita Giacaman

Richard J. Giannone

Travis Gibson

Ryland Giebelhaus

Rosario Gil

Jack A. Gilbert

Nicole M. Gilbert

Steven R. Gill

Sandra Gillespie

Christopher Gisriel

Silvia Giugliano

Erin Gloag

Jennifer L. Goff

Ryota Gomi

Cheng Gong

Jianping Gong

Júlia Brandão Gontijo

Sebastian Gonzalez

Victor Gonzalez

Benjamin Good

Emily C. A. Goodall

Heidi Goodrich-Blair

Friedrich Götz

Alexandre Gouzy

Marcin Grabowicz

Lone Gram

Nkrumah Grant

Joseph L. Graves

Tobias Grebe

Tiscar Grells

Paul Gribben

Joel S. Griffitts

Pranas Grigaitis

Jonathan Gropp

Denis Grouzdev

Felix Grünberger

Caroline Grunenwald

Rui Guan

Kirsten Raquel Guckes

Miguel Gueimonde

Sohini Guha

Emily Laura Gulliver

Fengbiao Guo

Linlin Guo

Alexey Gurevich

Juan-Carlos Gutiérrez

Jorge Gutierrez-Merino

Ana Gutiérrez-Preciado

Martin W. Hahn

Anna Hakobyan

Haley Hallowell

George Salem Hamaoui

Trinity L. Hamilton

Riadh Hammami

Jarrad Hampton-Marcell

Lijuan Han

Mei-Ling Han

Axel Kornerup Hansen

Shuai Hao

Janani Hariharan

Walter Harrington

Fumihito Hasebe

Amrita Hazra

Jia-Wei He

Brian P. Hedlund

Almut Heinken

Tory A. Hendry

Justus Hennecke

Michael Henson

Jennifer Heppert

Megan S. Hill

Yasmin Hilliam

Kelly Marie Hines

Ryuichi Hirota

Ying-Ning Ho

Paul Hodor

Monica Höfte

Camaron R. Hole

Johannes Holert

Emily B. Hollister

Karin Holmfeldt

Jan Homolak

Nazmul Hoque

Fujiang Hou

Qihui Hou

Shuwen Hou

Jennifer Houtz

Daniela Hozbor

Andrew J. Hryckowian

Anyi Hu

Canying Hu

Dalin Hu

Fupin Hu

Jun Hu

Junyi Hu

Renjing Hu

Sarah K. Hu

Furong Huang

Peng Huang

Gary B. Huffnagle

Ryan C. Hunter

Jillian H. Hurst

Inseong Hwang

Alexander P. Hynes

Federico Ibarbalz

Alexander N. Ignatov

Wakako Ikeda-Ohtsubo

Hidetoshi Inamine

Ikbal Agah Ince

Zahra Fatima Islam

William R. Jacobs

Alexander L. Jaffe

Pratik D. Jagtap

Soojin Jang

Scott A. Jarmusch

Paul R. Jensen

Weidong Ji

Zhongjun Jia

Huahua Jian

Jianfeng Jianfeng

Chao Jiang

Hongchen Jiang

Shuaiming Jiang

Jian-Yu Jiao

Mingliang Jin

Gongchao Jing

Sinu P. John

Korin Rex Jones

Marie Joossens

Vinayak Joshi

Igor B. Jouline

Feng Ju

Won Hee Jung

Chikara Kaito

Jessica K. Kajfasz

Christoph Kaleta

Alice Kane

Jea Woo Kang

Nagarajan Kannan

Enoch Kao

Antti Karkman

Lisa Karstens

Boran Kartal

Zuzanna Karwowska

Kazuyuki Kasahara

Takashige Kashimoto

Jonas Kasmanas

Jay D. Keasling

Johannes Keegstra

Chris Keneally

Tsuyoshi Kenri

Kristopher A. Kerns

Shahrbanoo Keshavarz Azizi Raftar

Mohammadali Khan Mirzaei

Arun S. Kharat

Devanshi Khokhani

Olga E. Khokhlova

Megan R. Kiedrowski

Silas Kieser

Joonhoon Kim

Min-Ju Kim

Seonghun Kim

Clare Louise Kirkpatrick

Raymond Kiu

Simon Klaes

Manuel Kleiner

Stefan Klumpp

Claudia Knief

Sho M. Kodera

Anthony J. Kohtz

Qing-Peng Kong

Konstantinos T. Konstantinidis

Tal Korem

Tomasz Kosciolek

Akihiro Koyama

Ariangela J. Kozik

Chana Kranzler

Laurent Kremer

Jens Kreth

Arianna I. Krinos

Erik Kristiansson

Marie Kroeger

Carsten Kröger

Kengo Kubota

Rakshak Kumar

Kyohei Kuroda

Kirsten Küsel

Svetlana Kutuzova

Brian H. Kvitko

Maria-Halima Laaberki

Alfredo Lagunas-Martínez

Leo Lahti

Kuei-Hung Lai

Andrew Laloo

Bert C. Lampson

Quentin Lamy-Besnier

Lefu Lan

Brian D. Lanoil

Janice E. Lawrence

Shuai Le

Raphael Ledermann

Elliot Michael Lee

Kihyun Lee

Vincent T. Lee

Mary Beth Leigh

Kyle R. Leistikow

Kgaugelo Edward Lekota

Paulo C. Lemos

Pok Man Leung

Uri Levine

Bailiang Li

Bei Li

Bingqing Li

Chunhao Li

Dayong Li

Fang Li

Fuyi Li

Guocai Li

Hongzhu Li

Huan Li

Jialing Li

Junhui Li

Rong Li

Ruichao Li

Tong-Pu Li

Wenjie Li

Xiang Li

Xiankun Li

Xiaoping Li

Yanyan Li

Zhe Li

Zhipeng Li

Tian Liang

Hanpeng Liao

Hebin Liao

Shen Jean Lim

Tong Wah Lim

Hao Lin

Miao-Hsia Lin

Xiangmin Lin

Morgan Lindback

Ning Ling

Di Liu

Fengping Liu

Haoran Liu

Ji Liu

Jia Liu

Jian-Hua Liu

Jinxin Liu

Liyun Liu

Long Liu

Xiang Liu

Xiaoyan Liu

Xingyin Liu

Xue Liu

Yang Liu

Yaoliang Liu

Yi-Fan Liu

Yongxin Liu

Zhihua Liu

Zhiru Liu

Melissa Bruckner Lodoen

Gamaliel López-Leal

Ramon Lorenzo-Redondo

Nicolas Louw

Adeline Loyau

Gabriel L. Lozano

Patricia Lozano-Zarain

Can Lu

Zhaoyu Lu

Zibin Lu

Liping Luan

Judith Carolina Lucena Nemirosky

Liliana Mercedes Ludueña

Mor N. Lurie-Weinberger

Tal Luzzatto-Knaan

Joshua Mark Lyte

Bo Ma

Chenchen Ma

Jincheng Ma

Qinhai Ma

Teng Ma

Yingfei Ma

Zhiyuan Ma

Daniel Machado

Rachel Mackelprang

Rohan Maddamsetti

Bhoomi Madhu

Elisse Magnuson

Finlay Maguire

John Makumbi

Mia Rose Maltz

Celia Manaia

Cristina Vieitez Manrique

Daniel K. Manter

Gina M. Many

Vanessa Marcelino

Maria L. Marco

Julia A. Maresca

Gabriele Margos

Joan Marquez-Molins

Jose Manuel Martí

Laura Martínez Alvarez

Irma Martinez-Flores

Celso Martins

Bruno Martorelli Di Genova

Eiji Masai

Charles Mason

Lewis Mason

Marios Mataragas

Amy Mathers

Alix Matthews

Ann G. Matthysse

Molly A. Matty

James McDonald

Margaret J. McFall-Ngai

Peter T. McKenney

Ane Hackbart de Medeiros

Daniel G. Mediati

Fernando Medina Ferrer

Conor Meehan

Lucas Meirelles

Joshua Chang Mell

Lingjie Meng

Guillaume Méric

Joshua K. Michener

Aram Mikaelyan

Aaron W. Miller

Christopher S. Miller

William R. Miller

Yusuke Minato

Nuno P. Mira

Oskar Modin

Andrew H. Moeller

Mohammad Mofrad

Rokhsareh Mohammadzadeh

Shadi Mohammed

Ipsita Mohanty

Wendy W. K. Mok

Elias Javier Mongiardini

Peter Kotsoana Montso

Jeremy P. Moore

Robert J. Moore

Rosario Morales-Espinosa

Ana Paula B. Moreira

Abigail Moreno

Andrey Morgun

Jiro F. Mori

Koji Mori

Ali Motahharynia

Daphne Moutsoglou

Kai Mu

Cécile Muller

Franziska Maria Müller

Marcelo Müller-Santos

Conrad W. Mullineaux

Vivek K. Mutalik

Dorota Muth-Pawlak

Jillian Myers

Kentaro Nagaoka

Satish K. Nair

Yusuke Nakade

Ernesto S. Nakayasu

David M. Needham

David E. Nelson

Shijulal Nelson-Sathi

Josef Neu

Michael L. Neugent

Gabriele Neumann

Irene L. G. Newton

Nguyen T. Q. Nhu

Yan Ni

Yueqiong Ni

Graeme William Nicol

Thomas D. Niehaus

Abigail Nieves Delgado

Mikeljon P. Nikolich

Henrik Nilsson

Kang Ning

Keita Nishiyama

Jozef I. Nissimov

Joshua D. Nosanchuk

Jozef Nosek

Minou Nowrousian

Takuro Nunoura

Spencer V. Nyholm

Bridget O’Banion

Toshihiro Obata

Nicholas H. Oberlies

Javier Ochoa-Repáraz

Mary O’Connell-Motherway

Erkison Ewomazino Odih

Masato Ogawa

Ole Andreas Økstad

Randall J. Olsen

William D. Orsi

Maghnus O’Seaghdha

John Osei Sekyere

Elizabeth A. Ottesen

Zhenlin Ouyang

Hazel Ozuna

Lars Pache

Jon E. Paczkowski

Stefano Pagliara

Utpal Pajvani

Jacob Palmer

Lauren D. Palmer

Tracy Palmer

Yue Pan

Surya Prakash Pandey

Suresh Panthee

Jason A. Papin

Eleftherios T. Papoutsakis

Elizabeth Parkinson

Ravikumar R. Patel

Sweta M. Patel

Kathryn A. Patras

Shyamal Peddada

Yu-Fang Pei

Zhangming Pei

Luiz Penalva

Bo Peng

Xuan-Xian Peng

Ariane L. Peralta

Leonardo Peraza-Reyes

Danilo R. Pérez-Pantoja

Kumar Perinbam

Hannes Peter

Eugen Pfeifer

Stefan Pflügl

Sujal Phadke

Vanessa V. Phelan

Oscar Quijada Pich

Amy C. Pickering

Alejandro Pironti

Lucia Pita

Jose R. Pittaluga

Georg Pohnert

Martin F. Polz

Olga Ponomarova

Ana Popovic

Kyle J. Popovich

Julie D. Pourtois

Arun Prakash

Carrie Pratt

Andrew Preston

Jeffrey Price

Arthur Prindle

Sambhawa Priya

Alexander J. Probst

Daniel Probst

Lita Proctor

Eugenii Protasov

Amy Pruden

Sumant Puri

Catherine Putonti

Junqing Qiao

Han Qu

Alisha Quandt

Lilliana Radoshevich

Roberta Rafetta

Sadia Isfat Ara Rahman

Sidra RahmatUllah Rahmatullah

Jean-Baptiste Raina

Huzefa A. Raja

Sasirekha Ramani

Kathryn M. Ramsey

Guanhua Rao

Xiancai Rao

Nikolai V. Ravin

Kasie Raymann

María Rebolleda Gómez

Hesper Rego

Barbara Rehermann

Olaya Rendueles-Garcia

Osbaldo Resendis-Antonio

Maria R. Esteban Torres

Nicole Ricker

Sebastián Riquelme

Line Roager

Erle S. Robertson

Jennifer Rocca

Christina E. Roche

Katherine Roche

Dmitry A. Rodionov

Jonathan Rodriguez

Miriam Rodriguez Fernandes

Luis Miguel Rodriguez-Rojas

Sanjay K. Rohaun

Kyle H. Rohde

Forest Rohwer

Benjamin R. K. Roller

Thibault Rosazza

Jason W. Rosch

Warren E. Rose

Benjamin Ross

Ella R. Rotman

Simon Roux

Shamik Roy

Piklu Roy Chowdhury

Zhi Ruan

Jamal Saad

Sandhini Saha

Jason W. Sahl

Christine E. Salomon

Dor Salomon

Steven L. Salzberg

Benedita Sampaio-Maia

Laura M. Sanchez

Gomathinayagam Sankaranarayanan

Joanne M. Santini

Fernandez Sántos Sanchez

Gaetano Santulli

John-Demian Sauer

Gary Sawers

Pauline Scanlan

Peter J. Schaap

Christopher W. Schadt

Patrick Schimmel

Patrick D. Schloss

Amy K. Schmid

Hannes Schmidt

Sebastian Schmidt

Thomas M. Schmidt

Wheaton L. Schroeder

Alison J. Scott

Nichollas E. Scott

Michelle R. Scribner

Ryan F. Seipke

Yuji Sekiguchi

Hoon Je Seong

Shiraz A. Shah

Abid Ullah Shah

Sadaf Shakoor

Anquan Shang

Nana Shao

Misheck Shawa

Jessica R. Sheldon

Scarlet S. Shell

Catherine D. Shelton

Bairong Shen

Weishou Shen

Yulong Shen

Zeyang Shen

Mang Shi

Pixu Shi

Zunji Shi

Masaki Shintani

Dylan Shropshire

Shantanu Shukla

Jonathan B. Shurin

Chandni Sidhu

Fitz Silao

Justin D. Silverman

Eby Mazini Sim

Magdalena Iga Skarżyńska

Mark S. Smeltzer

Wenke Smets

Clare M. Smith

Steph Smith

Caiti Smukowski Heil

Christian Sohlenkamp

Katarina Soltys

Fangfang Song

Hyun-Seob Song

Valerie Soo

Apollo Stacy

Christopher Staley

Brent A. Stanfield

Tom Stanton

Kenneth M. Stedman

Margaret I. Steele

Madison Elaine Stellfox

Bram W. G. Stone

Mason Stothart

Jan S. Suchodolski

Woo Jun Sul

Abby Sulesky-Grieb

Chaomin Sun

Haitao Sun

Jun Sun

Xuesong Sun

Zheng Sun

Zhihong Sun

Mangesh Suryavanshi

Vladimir Sustr

David Swainsbury

Jason B. Sylvan

Tatyana A. Sysoeva

Szymon P. Szafranski

Diana H. Taft

Rodrigo Gouvêa Taketani

Ralf Takors

Sudipta Talukder

James Tabi Tambong

Biao Tang

Himanshi Tanwar

Liang Tao

Siqi Tao

Dominique Tertigas

Malak Tfaily

Christopher Thibodeaux

Jonathan C. Thomas

Lance R. Thurlow

Peter Tiffin

Romie Tignat-Perrier

Sumeet Tiwari

Andrzej Tkacz

Julian Trachsel

Marie-Agnès Travers

Jai Justin Tree

Elizabeth Trembath-Reichert

Darren Trott

Kürsad Turgay

Massimo Turina

Jeffrey W. Turner

Marta Turon

Kevin Tveter

Lucas J. Ustick

Jose Utrilla

Jordan Vacheron

Filipa F. Vale

Luis E. Valentin-Alvarado

Riette Vanbiljon

Elodie Vandelle

Jan Roelof van der Meer

Hale Vanessa

Lynn Vanhaecke

Julia C. van Kessel

Geertje Van Keulen

Máté Vass

Christopher N. Vassallo

Roberto Vázquez

Siddarthan Venkatachalam

Ben Vezina

Sara Viera-Silva

Valeska Villegas-Escobar

Kinga Vojnits

Ueli von Ah

Jay Vornhagen

Catherine Ann Wakeman

Wan Gang Wan

Bo Wang

Chao Wang

Daoming Wang

Guoqing Wang

Hui Wang

Kunjie Wang

Le Wang

Min Wang

Mingbang Wang

Minxiao Wang

Nan Wang

Rui-Rui Wang

Xiaogang Wang

Yan Wang

Yang Wang

Yen-Wen Wang

Yongchao Wang

Yuehong Wang

Yuhao Wang

Zexin Wang

Zhang Wang

Liping Wang

Akaraphol Watcharawipas

Paula I. Watnick

Claudia Weber

Scott Weese

Zhong Wei

L. Weissman

Philipp Wendering

Darryl A. Wesener

Cheryl Whistler

Amber M. White

Rhys Thomas White

Wisnu Adi Wicaksono

Annegret Wilde

Floyd Wittink

Dagmar Woebken

Alan J. Wolfe

Benjamin Woolston

Nancy A. Woychik

Jing Wu

Ruonan Wu

Tong Wu

Kelly L. Wyres

Zuofu Xiang

Jianping Xie

Kabin Xie

Chenggang Xu

Jianping Xu

Juan Xu

Zhenjiang Zech Xu

Feng Xue

Huping Xue

Ting Xue

Fuhua Yan

Jiangwei Yan

Jinshui Yang

Julianne Yang

Liang Yang

Qinnan Yang

Ruifu Yang

Shihui Yang

Qingzhi Yao

Jinki Yeom

Laxmi Yeruva

Huabing Yin

Yi Yin

Sukhwan Yoon

Noha H. Youssef

Fangyou Yu

Lu Yu

Yunsong Yu

Joseph Zackular

Ruth N. Zadoks

Tiffany M. Zarrella

Vojtech Zarsky

Naiera Zayed

David Zeevi

Susan E. Zelasko

Lingbing Zeng

Karsten Zengler

Qixiao Zhai

Baojun Zhang

Bin Zhang

Chengcheng Zhang

Cuijing Zhang

Dengwei Zhang

Haifang Zhang

Jiachao Zhang

Jie Zhang

Lihan Zhang

Liqing Zhang

Peng Zhang

Suhua Zhang

Xinning Zhang

Xu Zhang

Yongyu Zhang

Yufeng Zhang

Zeng Zhang

Zhang Zhang

Chao Zhao

Chengcheng Zhao

Jian Zhao

Kun Zhao

Peng Zhao

Xin-Qing Zhao

Ying-Yong Zhao

Yashuang Zhao

Yanfen Zheng

Jin Zhong

Boyan Zhou

Heran Zhou

Jianliang Zhou

Jingyan Zhou

Xi Zhou

Xuguo Zhou

Yingshun Zhou

Zhichao Zhou

Bin Zhu

Lifeng Zhu

Yijun Zhu

Johannes Zimmermann

Erik R. Zinser

Haidong Zou

Huasong Zou

Ling Zou

Lingyun Zou

Cristal Zuniga

